# Improving the Automated Diagnosis of Breast Cancer with Mesh Reconstruction of Ultrasound Images Incorporating 3D Mesh Features and a Graph Attention Network

**DOI:** 10.1007/s10278-024-00983-5

**Published:** 2024-02-15

**Authors:** Sadia Sultana Chowa, Sami Azam, Sidratul Montaha, Md Rahad Islam Bhuiyan, Mirjam Jonkman

**Affiliations:** Faculty of Science and Technology, Charles Darwin University, Casuarina, NT 0909 Australia

**Keywords:** Breast tumor, Point cloud, Mesh, Feature extraction, Graph attention network (GAT), Ultrasound

## Abstract

This study proposes a novel approach for breast tumor classification from ultrasound images into benign and malignant by converting the region of interest (ROI) of a 2D ultrasound image into a 3D representation using the point-e system, allowing for in-depth analysis of underlying characteristics. Instead of relying solely on 2D imaging features, this method extracts 3D mesh features that describe tumor patterns more precisely. Ten informative and medically relevant mesh features are extracted and assessed with two feature selection techniques. Additionally, a feature pattern analysis has been conducted to determine the feature’s significance. A feature table with dimensions of 445 × 12 is generated and a graph is constructed, considering the rows as nodes and the relationships among the nodes as edges. The Spearman correlation coefficient method is employed to identify edges between the strongly connected nodes (with a correlation score greater than or equal to 0.7), resulting in a graph containing 56,054 edges and 445 nodes. A graph attention network (GAT) is proposed for the classification task and the model is optimized with an ablation study, resulting in the highest accuracy of 99.34%. The performance of the proposed model is compared with ten machine learning (ML) models and one-dimensional convolutional neural network where the test accuracy of these models ranges from 73 to 91%. Our novel 3D mesh-based approach, coupled with the GAT, yields promising performance for breast tumor classification, outperforming traditional models, and has the potential to reduce time and effort of radiologists providing a reliable diagnostic system.

## Introduction

Breast cancer is a prevalent disease which poses a threat to the women worldwide. It ranks as one of the three most fatal diseases for women due to the high mortality rate [1–3]. According to World Health Organization (WHO), more than 2.26 million new patients and 685 thousand deaths were reported in 2020 [4]. Early diagnosis of breast cancer can reduce the mortality rate and limit the tissue damage [5]. Two main categories of breast tumors are observed: benign (non-cancerous) tumors and malignant (cancerous) tumors. These tumors can be examined through various non-invasive imaging techniques, such as mammograms, ultrasounds, computed tomography (CT), and magnetic resonance (MR). Ultrasonography, a non-invasive method, is one of the most popular imaging modalities as it is painless, cheap, and radiation free [6–9]. The tumor patterns and characteristics can be distinguished through analysis of ultrasound images. However, this is very time consuming and the number of patients continues to increase.

Computer-aided diagnosis (CAD) systems to diagnose breast cancer can therefore support radiologists by providing improved and swift interpretation. Both benign and malignant tumors are the result of abnormal growth of the breast tissue but benign tumors do not spread to other areas of the body, have a regular oval shape, and usually consist of a smooth lesion [10]. Malignant tumor has micro lobulated or angular margins, indicating irregular pattern, and can spread to other organs if left untreated [11, 12]. To develop an effective CAD system, it is crucial to incorporate the characteristics of these tumors into an automated analysis system. Generally, the CAD systems utilize two-dimensional (2D) ultrasound images of the breast tumors, which cannot give insight in relevant features like the tumor volume [13] and overall tumor surface structure. On the other hand, three-dimensional (3D) imaging techniques such as CT, MR, and mammography have disadvantages like a high radiation dose or financial cost. Moreover, unlike 3D instruments, 2D imaging instruments are widely available in hospitals [14].

Graph-based models utilizing the radiologists’ decision strategy have proven themselves promising and reliable for automated diagnosis-based applications [15]. Therefore, in this study, we propose a breast tumor classification approach with a graph attention network (GAT) utilizing different clinically meaningful features extracted from the 3D mesh which is reconstructed from the 2D breast tumor region of interest (ROI). A breast tumor dataset, consisting of two classes benign and malignant, is utilized. The 3D mesh is generated from the 2D breast tumor ROI utilizing the point-e system proposed by Nichol et al. [16]. Ten medically relevant mesh features are extracted from the 3D mesh: centroid distance, minimum and maximum distance of mesh bounding box, surface area, volume, change of curvature, sphericity, anisotropy, eigen entropy, and farthest distance. Subsequently, feature selection techniques, such as minimum redundancy maximum relevance (MRMR) and analysis of variance (ANOVA) test, are conducted to assess the contribution of the features to the mesh classification where all the features exhibited adequate performance. For a comprehensive understanding on how the features of different classes showcase meaningful pattern and impactful for decision-making, a feature pattern analysis is experimented. A feature table is generated with dimensions of 445 × 12 containing ten feature columns, a target column, and a column consisting of unique IDs for the rows. Each row describes the characteristics of the mesh. A graph is generated incorporating the relationships between the rows of the feature table. Considering the rows as nodes, the Spearman correlation coefficient is used to find the edge between two correlated nodes. Two nodes are connected through an edge if the correlation score is greater than or equal to 0.7, so that only strong connections between the nodes are included. This threshold is determined through an experiment, where the proposed model’s performance with various threshold values ($\ge$ 0.5, $\ge$ 0.6, $\ge$ 0.7, $\ge$ 0.8, $\ge$ 0.9) is tested, and the model demonstrates its optimal performance with the 0.7 threshold value. The generated graph contains 56,054 edges and 445 nodes. An ablation study is conducted for seven variables, resulting in an optimized GAT model with the highest accuracy of 99.34%. Potential overfitting issues are assessed through k-fold cross validation. Results are presented through several performance matrices and statistical analyses. The performance of the proposed model is compared with ten machine learning models and one convolutional neural network (CNN) model. Finally, the performance of this study is compared with previous literature regarding the breast tumor classification. The major contributions of this study are listed below:

- 3D meshes are generated from the 2D breast tumor ROI which is able to provide more informative representation of the tumor.
- Instead of relying on the structure-based imaging feature, ten clinically relevant mesh features are extracted from the 3D mesh ROI.
- To classify the breast tumors, a GAT model is proposed, introducing graph representation of the mesh features which contributes greatly on the classification performance.
- To obtain a robust GAT model with higher accuracy, a seven-stage ablation study is performed; thus, the optimal configuration of the architecture is obtained.
- To further evaluate the GAT model’s performance, overfitting issue and performance consistency are assessed through several experiments.

## Related Work

In the field of early diagnosis of breast cancer, deep learning and machine learning approaches have made significant progress. Aljuaid et al. [17] employed a combination of deep neural networks (ResNet 18, ShuffleNet, and Inception-V3Net) and transfer learning on the publicly available BreakHis dataset to provide a computer-aided diagnosis technique for breast cancer categorization (both binary and multi-class). For both binary and multi-class classification, the ResNet model yielded the highest performance: 99.7%, and 97.81%, respectively. Chattopadhyay et al. [18] presented a state-of-the-art approach for classification of breast cancer where a dual-shuffle attention-guided deep learning model was developed. The proposed model included a channel attention mechanism. They evaluated the proposed model using the BreakHis dataset and obtained classification accuracies of 95.72%, 94.41%, 97.43%, and 98.1% for four different magnification levels, i.e., 40 × , 1000 × , 200 × , and 400 × , respectively. In the study of Abunasser et al. [19], breast cancer classification was carried out employing a transfer learning approach and a proposed CNN-based deep learning model, called BCCNN. The proposed model outperformed the transfer learning models with a classification accuracy of 98.28%. Jabeen et al. [20] proposed an automated system comprising image preprocessing, data augmentation, and deep learning for the classification of breast tumors. A pre-trained model, named EfficientNet-b0, was used to extract deep features and feature fusion was conducted utilizing a serial-based methodology. The experiments were conducted on two datasets, CBIS-DDSM and INbreast, with accuracies of 95.4% and 99.7%, respectively. Obayya et al. [21] developed a mathematical optimization algorithm with a deep learning-based method for classifying breast cancer using histopathological images (AOADL-HBCC). Utilizing image preprocessing, the proposed AOADL-HBCC technique achieved a maximum accuracy of 96.77%. In addition to traditional approaches, graph convolution networks with graph representation have been applied in the detection of breast cancer. Following this strategy, Yao et al. [22] created a model using a graph convolution approach that focuses on automatic identification and classification of calcification distribution patterns in mammographic images. The accuracy of the proposed model was 64.3%, and the area under the curve was 0.74%. An alternative method for classification of diseases is to create a point cloud from a 2D image and then generate a mesh from it, as this offers valuable information that can help with diagnosis. Sun et al. [23] developed a point cloud network LatentPCN which was used to reconstruct 3D surface models from calibrated biplanar X-ray data. LatentPCN transfers sparse silhouettes made from 2D images to a latent representation and this is used as the input to the decoder to create a 3D bone surface model. To assess the effectiveness of LatentPCN, experiments on two datasets were carried out. The mean reconstruction errors obtained by LatentPCN on these two datasets were 0.83 mm and 0.92 mm, respectively. Jia et al. [24] presented a pixel-wise sparse graph reasoning (PSGR) module for the segmentation of COVID-19-infected regions in CT images. The results show that the suggested PSGR module can successfully capture long-range dependencies, and the segmentation model can accurately segment COVID-19-infected regions. Liu et al. [25] presented a MIcrobial Cancer-association Analysis utilizing a Heterogeneous graph transformer (MICAH) to identify intra-tumoral cancer-associated microbial communities. Microbes’ phylogenetic and metabolic links were combined by the MICAH into a heterogeneous graph representation. The interaction between the intra-tumoral bacteria and cancer tissues was captured holistically using a graph attention transformer. Maken and Gupta [14] analyzed the computational techniques, specifications, and procedures for 3D reconstruction. They recommended further research of 3D reconstruction for X-ray images. Laumer et al. [26] proposed an automated approach which can infer a high-resolution, individualized 4D (3D plus time) surface mesh of the heart structures from 2D echocardiography video data. The inference of such shape models is a critical first step towards a tailored simulation that permits automatic evaluation of the heart chambers’ morphology and function. Hu et al. [27] developed a vision graph neural networks (ViG)-based pipeline that can classify breast ultrasound images. The accuracy of the ViG model was 100.00% for binary classification and 87.18% for multiclass classification. Ma et al. [28] presented a deep learning framework to store domain-specific transformations between the contours of the face and the bones for orthognathic surgery planning. To forecast the changes in the face and skeletal morphology, a bidirectional point-to-point convolutional network (P2P-Conv) was used. Their method outperformed state-of-the-art algorithms in terms of predicting face and skeletal shapes, according to experimental results with real-subject data.

Transforming 2D images into 3D representations and utilizing mesh representations is a novel and promising strategy in the field of disease detection and classification. The concept was introduced in several CAD-based studies; however, to the best of our knowledge, no one has yet attempted to classify breast ultrasound images with this strategy. Traditionally, breast ultrasound images analysis is conducted in 2D. This has drawbacks because it only shows the breast tissue slice by slice, thus missing out on important spatial information. A more comprehensive understanding of the structure of breast tissue can be attained by transforming 2D images into 3D representations, opening up new possibilities for improved detection and classification. By converting traditional 2D imaging data into a more comprehensive and informative 3D format, this cutting-edge method presents a novel viewpoint. In this study, the concept is utilized with graph representation of clinically relevant mesh features of ultrasound images. In most of the previous studies, 2D imaging features were employed with a classification approach using traditional machine learning methods and the clinical relevance was not further investigated. This study also differs from previous research by incorporating graph model-based classification based on established node to edge relationships.

## Materials and Methods

### Dataset

**Fig. 1:**
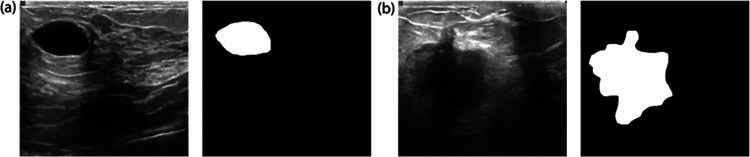
Samples of breast ultrasound images. **a** Image of a benign tumor and **b** image of a malignant tumor with their corresponding ground truths

A publicly available dataset [29] is used in this study. It includes 780 breast ultrasound images of 600 female patient’s aged between 25 and 75 years. The images are in PNG format and categorized into benign, malignant, and normal classes, containing 437, 210, and 133 images, respectively. Ground truth masks for the ROIs were provided for the benign and malignant classes. As this study is focused on breast tumor classification, the 647 ultrasound images for the benign and malignant classes are used. Sample images of benign and malignant images with their respective masks are shown in Fig. 1.

### Methodology

**Fig. 2:**
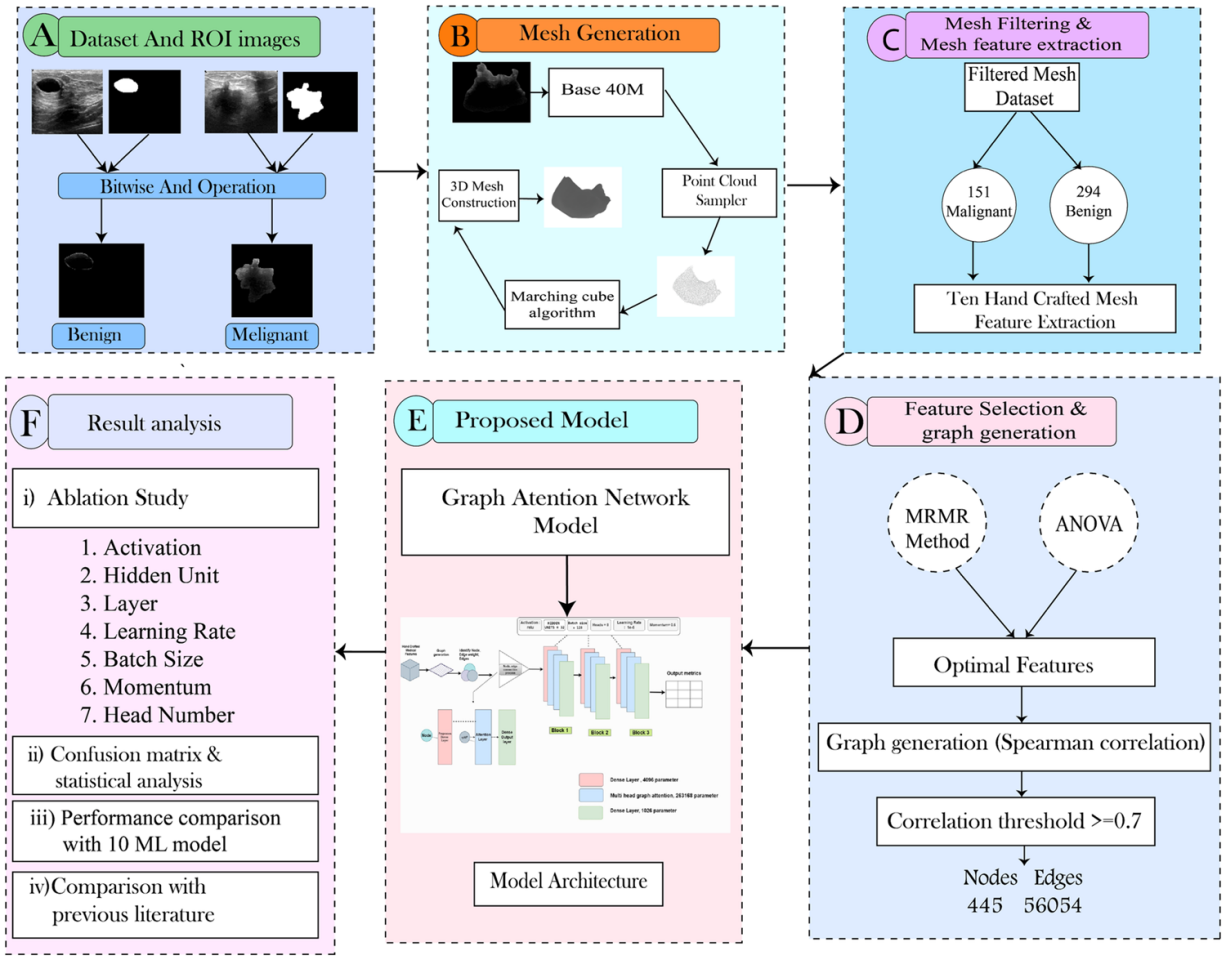
Overview of the methodology pipeline for breast tumor classification (**A**: dataset and ROI extraction, **B**: mesh generation, **C**: mesh filtering and mesh feature extraction, **D**: feature selection and graph generation, **E**: model selection, **F**: analysis of results)

The methodology pipeline of this study is shown in Fig. 2.

The first step (A) is tumor ROI segmentation, using a bitwise AND operation between the raw images and the provided ground truth masks. This ensures that only relevant parts are taken into consideration for further analysis. The objective of step B is to create a 3D mesh from the tumor ROI. For this task, the segmented images are fed into the Base40M model, which creates a dense point cloud of the ROI using an additional point cloud sampler model. The mesh is then generated from a regression-based model and a marching cube algorithm. In step C, a filtering process is carried out, taking into consideration that some meshes may differ significantly from their 2D image because of the lack of accurate depth information in the raw images. To ensure higher quality 3D meshes of the tumor ROI, images are selected by hand and only the finest meshes are selected. After filtering, 294 images of benign and 151 images of malignant tumors are selected out of 437 benign and 210 malignant images, respectively. Then, ten meaningful features are extracted from the filtered mesh dataset. These features provide information regarding the characteristics of the tissues. In step D, two methods are used for feature selection: ANOVA and MRMR. These techniques enable the identification of the most relevant and informative features, in order to ensure correctness and reliability of further analysis. Then, a feature pattern analysis is done to understand the distinctive characteristics of each class even better. After establishing correlations between the chosen features using Spearman correlation threshold 0.7, a graph is constructed, resulting in 445 nodes and 56,054 edges. In step E, a GAT model is developed and the graph is fed into the model. To enhance the model’s performance, an ablation study is conducted with seven parameters: the activation function, hidden unit, number of layers, learning rate, batch size, momentum, and number of heads. In the final step, F, a comprehensive analysis of the results is done. A confusion matrix, k-fold cross validation, statistical analysis, and performance comparison with ten machine learning models, as well as a comparison with previous studies, are presented. By using this methodology, this study provides an analysis of breast ultrasound classification and may provide insight and expand our understanding of the features and abnormalities of breast tissue.

### Mesh Dataset Generation Using Point-e Network

**Fig. 3:**
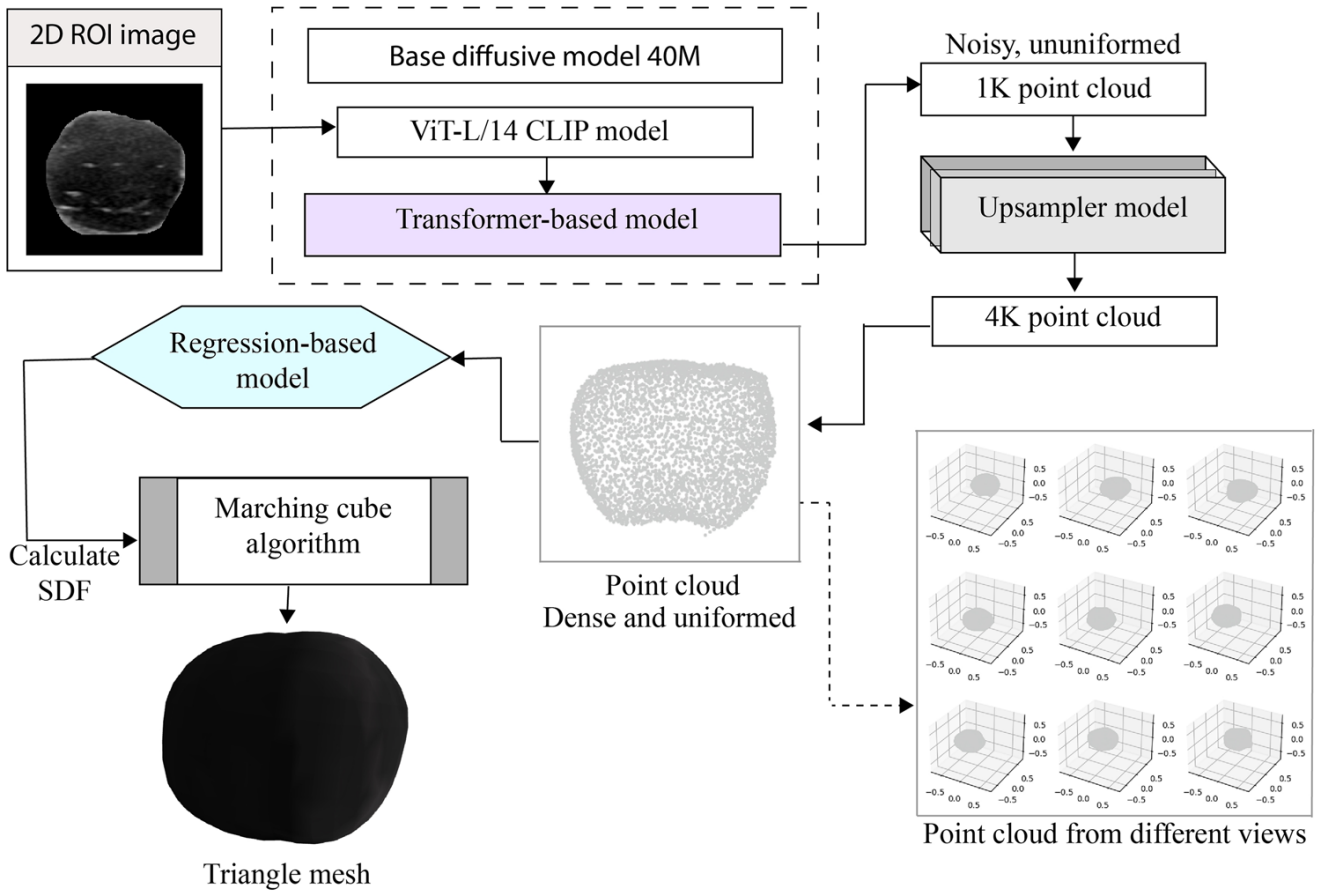
Mesh generation from the 2D breast ROI

The breast tumor ROI is extracted using a bitwise AND operation between the ultrasound image and the ground truth. With the breast tumor ROI’s, a mesh dataset is generated by the point-e system. The mesh is generated based on the RGB point cloud of the 2D breast ROI using a regression-based approach. A stack of diffusion models in accordance to the study of Nichol et al. [16] is used for the conversion of 2D image to a 3D point cloud. Figure 3 shows the mesh generation procedure from 2D tumor ROI images.

The 40 M diffusive model is utilized for the point cloud generation of the breast ROI. The image is fed into a frozen pretrained contrastive language-image pre-training (CLIP) model, named ViT-L/14 CLIP, a large vision transformer model consisting of 14 layers, for the generation of the point cloud. The output of the ViT-L/14 CLIP model is then fed into a transformer-based model which makes a prediction of a set of possible conditions and noised point cloud.

To deal with noisy and non-uniform point clouds, generated through the transformer, an additional up-sampling model is used to get a uniform, high-resolution, and dense point cloud. The architecture of the up-sampling model is same as the architecture of the base 40 M diffusive model. The base model first generates a point cloud at low resolution, of 1 k points. The 40 M diffusive model is conditioned based on the low-resolution point cloud, serving as an up-sampling model. So, the output point clouds of base diffusive model are then up sampled with the up-sampling model, generating 3 k points in addition. Thus, a dense uniform point cloud object is constructed with 4 k points. The objective of the base diffusive model is to only generate low-resolution point cloud that will hold an overall structure of the given input. Subsequently, a high resolution-based point cloud is generated through the up-sampling model with the structural information obtained from low-resolution point cloud [16, 30]. Then, utilizing a regression-based model, the signed distance field (SDF) of the point clouds are calculated and a marching cube algorithm extracts the meshes from the points. The regression-based model is basically a regression forest-based method to predict the location of a grid point within a 3D space that is essential to compute the SDF. The signed distance estimates each point distance of the point cloud from the 3D surface boundary. A positive SDF value defines the point is outside of the 3D surface boundary and a negative SDG means the point is inside the 3D surface boundary. The SDF for a point *p* can be calculated from following equations [31]:

1 $${\pi }_{s}\left(p\right)=arg{ min}_{{p}{\prime}}|{p}{\prime}-p|$$

2 $${\Delta }_{s}\left(p\right)=p-{\pi }_{s}\left(p\right)$$

3 $${d}_{s}\left(p\right)=|{\Delta }_{s}\left(p\right)|$$

4 $${s}_{s}\left(p\right)={d}_{s}\left(p\right)sgn({\Delta }_{s}\left(p\right)\bullet {n}_{s}\left({\pi }_{s}\left(p\right)\right))$$

Here, ${\pi }_{s}\left(p\right)$ denotes the projection (location within a 3D space) of the point *p* to the surface *s*. The projection difference between the point *p* and its projection ${\pi }_{s}\left(p\right)$ is outlined by ${\Delta }_{s}\left(p\right)$. Then, the unsigned distance ${d}_{s}\left(p\right)$, for the point *p*, is calculated for the surface *s*. Lastly, the signed distance ${s}_{s}\left(p\right)$ is estimated by evaluating the agreement between the ${\Delta }_{s}\left(p\right)$ and the normal at *p* that is projected onto the surface, represented as ${n}_{s}\left({\pi }_{s}\left(p\right)\right)$. After calculating the signed distance for each point of the point cloud, the process of 3D surface reconstruction, also known as isosurface, is carried out by the marching cube algorithm. The marching cube algorithm generates triangles of the signed distance point in a voxel grid to approximate the target isosurface [32]. Finally, the 3D point cloud obtained from the 2D image is converted into a solid mesh.

Speckle noise in ultrasonic images reduces tissue boundaries, produces deceptive structures, and makes it difficult to segment and classify them. For which the tumor’s depth, edges, and pixel distributions cannot be identified in some cases [33]. Constructing a 3D view from an image that lacks comprehensive shape and edge information is a complex task as it needs to predict the incomplete parts effectively [34–36]. The distortion caused by speckle noise may lead to difficulties in generating a coherent 3D representation and become a potential cause of poor and sparse point cloud generation. Furthermore, estimating mesh from such sparse and noisy point cloud is challenging. A noisy point cloud has more likelihood to adversely impact the accurate computation of the SDF [37, 38] that cannot represent the actual object surface and disrupt the mesh generation process, resulting poor mesh structures like mesh holes (the unwanted gaps in the surface of a triangular mesh). These mesh holes affect the actual topology structure of the tumor mesh and mislead the breast tumor’s classification task [38]. In order to identify the deceptive structured meshes, an experiment is conducted, quantifying the percentage of mesh holes presented in a mesh. The quality of medical data can significantly impact on the diagnosis process [39, 40]. As this study diagnoses the breast tumor with medically significant mesh features, it is considered essential to work with high-quality meshes. So, meshes with more than 10% of their structure compromised by mesh holes are considered bad tumor meshes and excluded from the further diagnosis process. This criterion is consistently applied to all meshes during the mesh removal process, ensuring transparency and consistency in the identification of meshes with structural compromises. This criterion upholds a high standard of mesh quality, reducing the probability of uncertainties due to structural compromise that could potentially affect the precision of the breast tumor diagnosis. The investigation seeks to enhance the robustness of the diagnostic process by using this exclusion threshold, emphasizing the significance of meticulous attention to mesh quality in medical imaging applications.

**Fig. 4:**
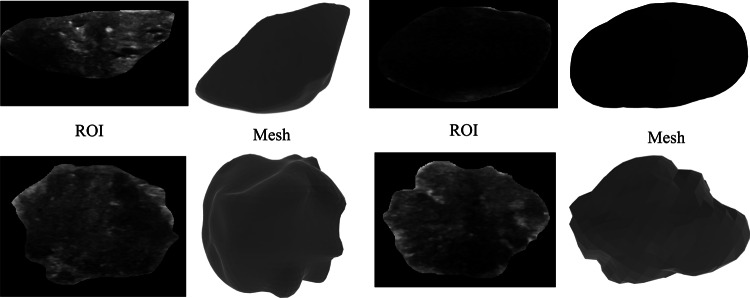
Tumor ROIs with their corresponding meshes

Hence, after generating the mesh dataset for 647 images, some images are removed due to the presence of deceptive structures, resulting in a dataset of 445 meshes of 294 benign tumors and 151 malignant tumors. This updated mesh dataset is used in the further classification procedures. Some sample mesh structures generated from the tumor ROI are shown in Fig. 4.

### Feature Extraction

Clinically relevant features are superior for the classification of breast tumors. Benign and malignant tumors differ in shape, structure, volume, and tumor orientation pattern [41]. We propose a set of 10 mesh features to differentiate malignant and benign characteristics. The features are separated into two categories, structural-based and distance-based.

#### Structural-Based Features

The structural-based mesh features include curvature, volume, surface area, eigen entropy, anisotropy, and sphericity. Most of the mesh features are calculated based on eigenvalues. The covariance features of anisotropy, centroid, and eigen entropy of the tumor ROI mesh tensor can be obtained from the normalized eigenvalues (${\lambda }_{i}$). The covariances for the mesh triangle are defined as ${\lambda }_{1}$, ${\lambda }_{2}$, and ${\lambda }_{3}$ and the order is ${\lambda }_{1}$ ≥ ${\lambda }_{2}\ge {\lambda }_{3}$  ≥ 0 [42, 43]. A description of the structural-based features is given in Table 1.

**Table 1:** The structural-based mesh features

Features	Descriptions	Equations
Curvature (*C*) [42, 44]	A measure of the local curve of the mesh surface calculated based on the angles of the mesh triangles	$C={\text{mean}} (\frac{{\lambda }_{3}}{{\lambda }_{1}+{\lambda }_{2}+{\lambda }_{3}})$
Volume (*V*) [45]	The volume of the space within the enclosing surface triangles	$V={\text{mean}} (\sum \frac{1}{6}\left({v}_{1}\times {v}_{2}\right)\bullet {v}_{3})$ Here, ${v}_{1}, {v}_{2}, {v}_{3}$ are the vertices of the mesh triangles
Surface area (*Sa*) [45]	The total area covered by the mesh triangles	$Sa={\text{mean}} (\sum \frac{1}{2}\left({v}_{2}-{v}_{1})\times {(v}_{3}-{v}_{1}\right))$
Eigen entropy (*E*) [42]	The irregularity of the tumor’s surface triangles	$E={\text{mean}} (\sum_{i=1}^{3}{\lambda }_{i}{\text{ln}}({\lambda }_{i}))$
Anisotropy (*A*) [42, 43]	The directional distribution of the points and the uniformity presented of the tumor mesh surface	$A={\text{mean}} (\frac{{\lambda }_{1}-{\lambda }_{3}}{{\lambda }_{1}})$
Sphericity (*S*) [46]	A measure of how close the tumor’s mesh is to a sphere. It is a measure of the mesh roundness	$S=\mathrm{mean }(\frac{{(\pi )}^{{~}^{1}\!\left/ \!{~}_{3}\right.} {(6V)}^{{~}^{2}\!\left/ \!{~}_{3}\right.}}{Sa})$

The malignant tumor structure often has spiculated margin and is non-uniform while the benign tumor’s structure has a smooth and consistent pattern [10, 11]. The structural mesh features usually have a higher value for malignant tumors.

#### Distance-Based Features

**Fig. 5:**
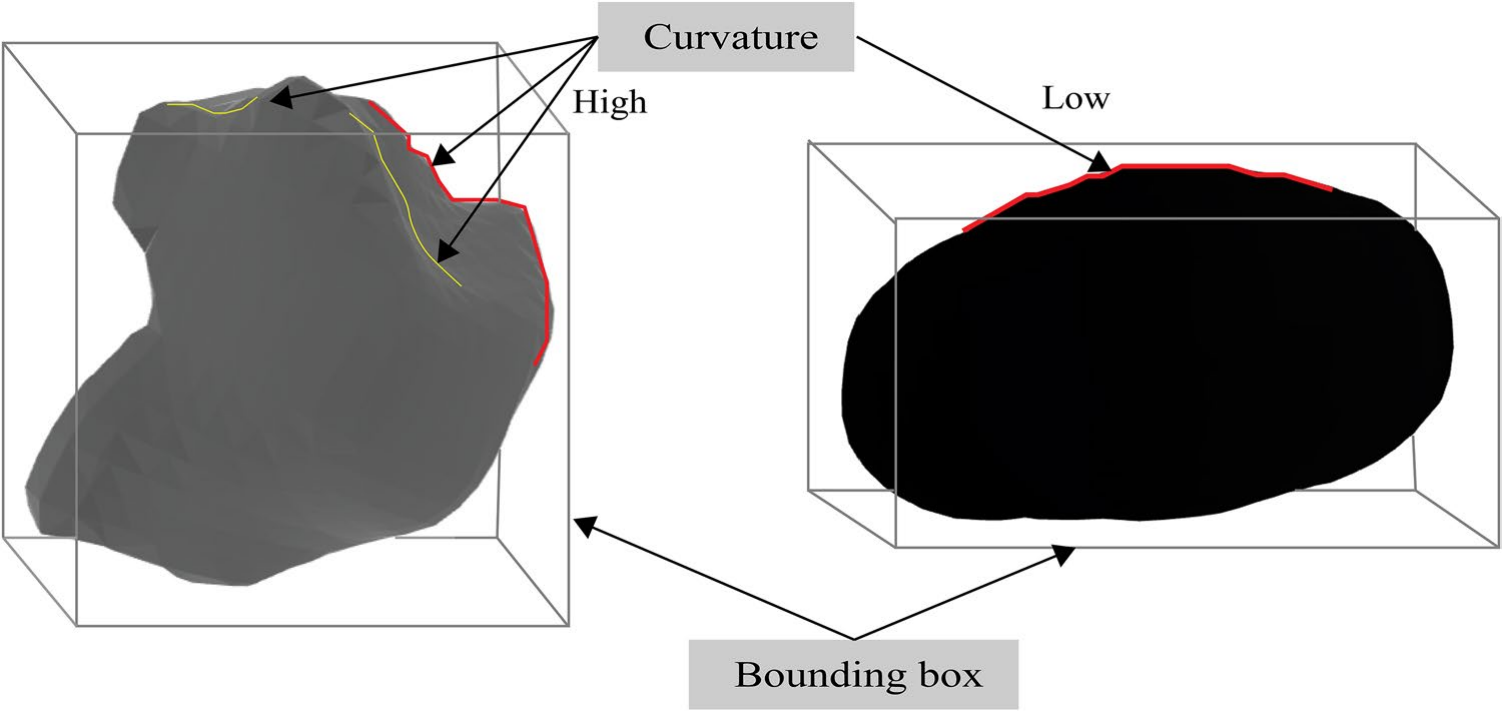
Visualization of the mesh with bounding box and curvature

The distance-based mesh features are based on the distance between two mesh points. Minimum distance of the bounding box, maximum distance of the bounding box, farthest distance, and centroid distance are considered distance-based features. The minimum bounding box distance (BB min distance) and maximum bounding box distance (BB max distance) represent the shortest and longest distance between points of the mesh and the bounding box. The farthest distance represents the maximum distance between two vertices of the mesh. The centroid distance refers to the Euclidean distance between the center of the mesh object and the mesh points. The centroid represents the central location of all the mesh faces. Because malignant tumors have curvy, irregular, and non-uniform structure, the values of distance-based features are usually higher for the mesh of a malignant tumor. Since the benign tumor mesh is smooth and uniform, and angles between the mesh triangles are minimal, the distance-based scores are lower for benign tumors except the centroid distance feature. Figure 5 shows a visualization of mesh feature analysis with the bounding box and the curvature.

### Feature Pattern Analysis

In this experiment, a feature pattern analysis is focused with the objective to compare the differences between the feature values of benign and malignant classes. The analysis is carried out by individually computing the mean of features for each class. Table 2 represents the outputs of feature pattern analysis.

**Table 2:** Feature pattern analysis (individual and mean feature value)

Image/mesh	Sphericity	Anisotropy	Eigen entropy	Curvature	Farthest distance	Centroid distance	BB min distance	BB max distance	Volume	Surface area
For malignant meshes										
1	0.027	0.316	0.135	0.528	1507	0.235	0.602	0.691	0.151	0.815
2	0.025	0.315	0.129	0.604	3299.77	0.183	0.613	0.706	0.324	2.677
3	0.028	0.357	0.128	0.672	2482	0.04	0.702	0.729	0.134	1.705
4	0.022	0.298	0.098	0.593	2449.92	0.106	0.576	0.691	0.091	1.107
5	0.028	0.329	0.137	0.607	1894	0.257	0.623	0.761	0.152	1.436
Average threshold for malignant meshes (malignant → ${\mu }_{M}$)										
${\mu }_{M}$(70)	0.027	0.314	0.124	0.592	1990.38	0.26	0.609	0.705	0.143	1.489
${\mu }_{M}$(90)	0.025	0.305	0.101	0.601	1859.57	0.279	0.627	0.697	0.143	1.426
${\mu }_{M}$(110)	0.026	0.319	0.133	0.579	1927.83	0.278	0.616	0.703	0.14	1.476
${\mu }_{M}$(130)	0.027	0.321	0.138	0.603	2013.21	0.27	0.627	0.71	0.151	1.561
${\mu }_{M}$(150)	0.028	0.325	0.129	0.607	2061.26	0.257	0.625	0.715	0.154	1.579
For benign meshes										
1	0.012	0.177	0.042	0.238	1338	0.297	0.247	0.624	0.012	0.637
2	0.013	0.173	0.038	0.285	961	0.409	0.468	0.627	0.057	0.32
3	0.014	0.203	0.063	0.304	998	0.343	0.765	0.649	0.029	0.703
4	0.013	0.181	0.054	0.275	870	0.414	0.526	0.628	0.055	0.637
5	0.008	0.159	0.039	0.228	612	0.465	0.358	0.647	0.012	0.291
Average threshold for benign meshes (benign → ${\mu }_{B}$)										
${\mu }_{B}$(70)	0.013	0.183	0.055	0.28	809.44	0.414	0.498	0.636	0.058	0.607
${\mu }_{B}$(90)	0.014	0.188	0.057	0.286	866.9	0.41	0.502	0.628	0.052	0.659
${\mu }_{B}$(110)	0.013	0.187	0.057	0.3	862.79	0.396	0.497	0.629	0.055	0.692
${\mu }_{B}$(130)	0.014	0.181	0.057	0.302	898.55	0.402	0.502	0.633	0.053	0.681
${\mu }_{B}$(150)	0.013	0.184	0.056	0.286	881.89	0.399	0.495	0.631	0.053	0.675
Average threshold for all meshes (${\mu }_{B}$ and ${\mu }_{M}$)										
${\mu }_{M}$(151)	0.028	0.324	0.139	0.607	2053.83	0.268	0.625	0.714	0.153	1.575
${\mu }_{B}$(294)	0.013	0.184	0.054	0.283	897.86	0.396	0.492	0.631	0.053	0.674

In Table 2, five random mesh features from malignant and benign classes are shown to examine the actual distribution pattern of their features and emphasize the differences. Then, the average (*μ*) values of each feature for both classes are computed. The process involves six different cases, where in first five scenarios, 70, 90, 110, 130, and 150 randomly selected feature values are taken and the average values are counted for each scenario. Finally, in the last case, the average is calculated considering all the mesh feature values of each class, respectively. By analyzing the average values derived from each case study, a difference is found for malignant and benign features which can cluster them into two groups according to the feature value distribution patterns.

It has been observed that the feature values of sphericity for malignant is much higher compared to benign tumors. As benign tumors are mostly round or oval shaped [47], they have a higher likelihood of closely resembling a sphere and maintain a lower sphericity value. As a result, the average sphericity values for benign tumors are approximately half that of malignant tumors. Besides, the values of the anisotropy and eigen entropy features are notably higher in malignant tumors than in benign tumors. This disparity denotes the presence of non-uniformity in malignant tumors and an explicit uniformity in benign tumors. The curvature, farthest distance, BB min distance, and BB max distance features show greater values for malignant tumor than benign. Conversely, the values of centroid distance are higher for benign as they contain compact cell arrangement [48] and lead a larger centroid distance while the scattered cell pattern and heterogeneity [49] of malignant tumors lead smaller centroid distance value. These outcomes evidenced the pattern complexity of malignant tumors that contain poorly structured and curvy lesion. Tumor size can be a prominent discriminator between benign and malignant [50]. Analyzing the volume and surface area features, it is obvious that malignant tumors are greater in shape than benign. The discussion aligns with the observation of feature pattern analysis. Thus, the experiment is able to discover important insights into the distinctive feature patterns of both groups.

### Feature Selection

Feature extraction is used to obtain the essential markers of an object, whereas feature selection selects the most informative and relevant features for the classification task. Using these features, a cost-effective and fast system performance can be achieved, while reducing overfitting and enhancing interpretability [51]. A univariate feature selection method, ANOVA, and a multivariant feature selection method, MRMR, are applied in this study to assess the significance of the separate features, filter out the irrelevant features and get the optimal feature combination, and evaluate the differences between the two classes [52, 53]. The result of the ANOVA test is given in Table 3.

**Table 3:** Result analysis of ANOVA test

Features	*F* value of ANOVA test	*p* value of ANOVA test
Centroid distance	75.9494	5.868e − 17
Surface area	131.017	9.388e − 27
Volume	80.750	7.442e − 18
Curvature	156.676	5.457e − 31
Sphericity	88.744	2.499e − 19
Anisotropy	121.555	3.835e − 25
Eigen entropy	110.394	3.317e − 23
Farthest distance	106.415	1.664e − 22
BB min distance	59.167	9.469e − 14
BB max distance	46.7739	2.655e − 11

A difference between separated groups is considered significant if the *p* value is less than 0.5 [54]. From Table 3, we can see that all *p* values are extremely low, indicating a large difference between the compared groups and validating that these features are suitable for the classification task.

**Fig. 6:**
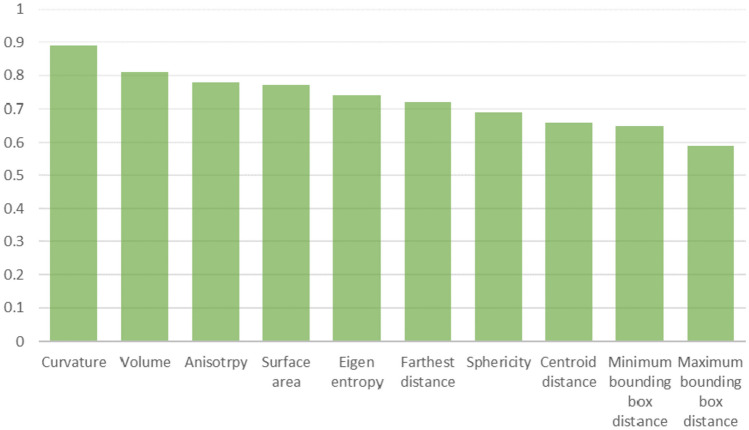
MRMR ranking the mesh features based on their relevance with target groups

The MRMR feature selection technique is used to rank the features based on their relevance for the target groups [55]. Figure 6 shows the MRMR ranking for the mesh features.

All features score more than 50%, see Fig. 6, revealing a strong correlation between the features and the target groups. All features perform well for both ANOVA and MRMR, verifying themselves appropriate for classifying the breast tumor mesh.

### Graph Dataset Generation

A graph dataset is generated from the feature table to understand the profound semantic structure among meshes within the same classes. The dimensions of the feature table are 445 × 12, with ten feature columns, one target column, and one column consisting of a unique ID for each individual row. A row is a characteristic representation of a specific tumor mesh and the column unique ID of each row is only used to assign node numbers and identify graph nodes. The rows are considered graph nodes and the relationship between the rows are considered as graph edges. For instance, the relationship between first raw (unique ID = 1) and second row (unique ID = 2) is determined where the edge will be drawn from the source node “1” to the target node “2.” The Spearman correlation coefficient is calculated to estimate the relationships between rows excluding the target column and unique ID column as they do not contain any information of a specific mesh feature. This results in a connected graph dataset where all nodes are linked. A graph is generated based on a 0.7 correlation threshold which means that if the correlation score is equal or above 0.7, an edge (${e}_{k};\mathrm{ where} k\in \mathrm{1,2},\mathrm{3,4},\dots N)$ is constructed between two nodes (${n}_{i},{n}_{j};\mathrm{ where} i,j\in \{\mathrm{1,2},\mathrm{3,4},5,\dots 445\} {\text{and}} i\ne j)$. Table 4 shows the correlation pattern of the graph dataset.

**Table 4:** Graph dataset containing source node, target node, and correlation between two nodes

Edge number	Source node	Target node	Correlation
1	1	2	0.97
2	1	3	0.98
3	1	4	0.85
4	1	5	0.95
5	1	6	0.97
…	…	…	…
56,050	441	442	0.99
56,051	441	443	0.90
56,052	441	444	0.92
56,053	442	443	0.88
56,054	442	444	0.90

The graph dataset in Table 4 displays the source and target nodes along with their correlation scores. The dataset outlines the procedure followed to generate the edge based on the value of the Spearman correlation coefficient. The table demonstrates that edge “1” links nodes “1” and “2” with a correlation score of 0.97, presenting a strong relationship between the nodes. The graph dataset of Table 4 reflects the connection between meshes offering a powerful representation of the degrees of similarity and dissimilarity that can fluctuate in accordance to their ten key feature values and corresponding classes. It is observed that 56,054 edges are created among 445 nodes, where 11,325 edges are built between nodes of malignant class and 42,778 edges are generated between nodes of benign class, indicating the presence of complex and substantial connections between nodes. Remaining 1951 edges are generated between benign and malignant classes because certain benign and malignant breast tumor lesions may exhibit high degree of resemblance [56]. This dataset not only represents the structural relationships between meshes but also captures the nature and interactions of these relationships. It provides a comprehensive topological representation that preserves strong neighboring relations [57]. Thus, the flexibility for in-depth analysis can be achieved through the utilization of a graph data structure, which enables to explore the detailed underlying relationships. This approach facilitates a more profound understanding of the interactions between different features and classes, enhancing the efficacy of classification algorithms.

### Model

There have been several attempts to adapt neural networks to deal with arbitrarily structured graphs. Sequence-based approached nowadays make use of attention processes [58]. One advantage of the attention mechanisms is that they make it possible to handle inputs of different sizes while concentrating on the information that is most important for making decisions. Self-attention and intra-attention are terms used to describe an attention process that computes a representation of a single sequence [59]. In this classification approach of breast ultrasound image using 3D mesh features, a graph-based GAT model is introduced and this model is optimized with ablation study where seven parameters of the model are modified. The ablation study results can be found in Table 5.

#### Graph Attention Layer

The building block layer used to create arbitrary graph attention networks is described in this section, along with its theoretical and practical advantages and potential applications in neural graph processing.

**Fig. 7:**
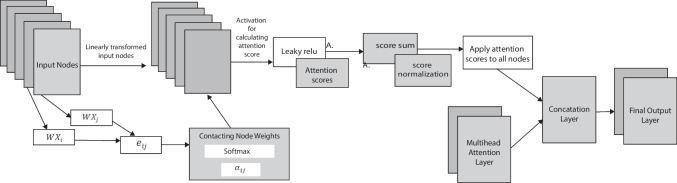
Mechanism graph of attention layer processing

This section will outline the GAT model’s single preprocessing layer for the graph attention mechanism. The entire processing method is described in Fig. 7.

The input layer is the set of nodes which represent the features. Nodes $\left\{X=X1,X2,X3\dots \dots {X}_{n}\right\}, {X}_{i}\in R$ where $X$ refers to the feature values and *R* represents all real numbers. The edges can be represented as ${e}_{ij}=a(W{X}_{i},W{X}_{j})$ where *W* is the weight of the edge and ${X}_{i},{X}_{j}$ are the relational nodes. $W{X}_{i}$ and $W{X}_{j}$ are the node weights which are concatenated by the SoftMax function as ${\alpha }_{ij}$ [59]. The equation is:

5 $${\alpha }_{ij}=Softmax\left({e}_{ij}\right)=\frac{{\text{exp}}({e}_{ij})}{\sum_{k\in {N}_{i}}{\text{exp}}({e}_{ik})}$$

An attention score is computed using a set of concatenation node weights. The Leaky ReLU activation function, a variant of the ReLU activation function, is used in the calculation of the attention score. After calculating the attention scores for all nodes, they are summed using a mathematical function called “math.bin Count.” To ensure that the attention scores are in a consistent range and avoid issues with scale, the attention scores are normalized during further preprocessing. Moreover, the next step is applying the normalized attention scores to all nodes. This procedure effectively emphasizes specific nodes based on their significance by multiplying each node’s concatenated weights by its associated normalized attention score. A multi-head attention layer is then applied as the final layer of preprocessing. The computation of multiple sets of attention weights, each referred to as a “head,” constitutes the multi-head attention mechanism. These attention heads provide the opportunity to learn various patterns by capturing different interactions between the nodes. Concatenating the outputs of these attention heads creates a final layer output that combines the information gained from different characteristics of the input nodes.

#### GAT

**Fig. 8:**
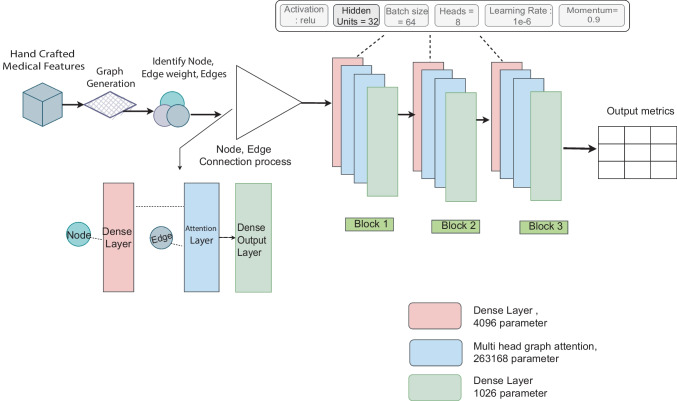
Proposed model architecture after ablation study

Our GAT model is a combination of the two dense layers and two multi-head graph attention layers based on the multi-head graph attention mechanism. Figure 8 shows the optimized architecture of GAT model after ablation study.

The proposed model utilizes the features and transforms them into a complex graph representation. The nodes, edges, and their weight all combine to create the input for our model. After node edge connection, the nodes are connected with the dense layer and the edges are connected with the attention later. A dense layer with 4096 parameters, a multi-headed graph attention layer with 263,168 parameters, and a final dense layer with 1026 parameters are part of each of the three blocks. An ablation study is being carried out to optimize the model, investigating the effects of various elements on the performance.

#### One-Dimensional CNN Model

Sequential data analysis utilizes a one-dimensional (1D) CNN model. Convolutional layers, pooling layers, and one or more fully linked layers are all components of the 1D CNN architecture. In order to extract relevant features from the input data, the convolutional layers incorporate a number of filters, and to reduce the number of parameters in the network, the pooling layers down sample the output of the convolutional layers [60]. The fully linked layers generate predictions using the obtained features.

## Results

Our proposed model is evaluated using several performance metrics, training accuracy, test accuracy, precision, recall, F1 score, negative predictive value (NPV), false positive rate (FPR), false discovery rate (FDR), false negative rate (FNR), and Matthew’s correlation coefficient (MCC). These metrics are calculated based on the true positives (TP), true negatives (TN), false positives (FP), and false negatives (FN) values acquired from the confusion matrix. An ablation study is done and the performance of the model is compared with previous literature and ten machine learning models.

### Ablation Study

Seven ablation experiments are carried out to optimize the performance of the proposed GAT model. In each stage, the configuration resulting in the highest test accuracy is chosen for further experimentation. The outcome of the seven ablation experiments is listed in Table 5.

**Table 5:** Ablation study for GAT model optimization

Activation function			
No	Activation function	Test accuracy (%)	Average time per step (second)
1	elu	96.05	29 s
2	relu	96.71	29 s
3	tanh	89.54	29 s
Hidden units			
No	Hidden units	Test accuracy (%)	Average time per step (second)
1	64,64	92.11	27 s
2	32,32	97.37	22 s
Attention layers			
No	No. of attention layers	Test accuracy (%)	Average time per step (second)
1	2	97.37	25 s
2	3	96.05	27 s
3	4	98.03	28 **s**
Learning rates			
No	Learning rate	Test accuracy (%)	Average time per step (second)
1	0.0001	93.42	27 s
2	0.00001	98.03	29 s
3	0.000001	98.68	27 s
Batch size			
No	Batch size	Test accuracy (%)	Average time per step (second)
1	32	98.03	57 s
2	64	98.68	34 s
3	128	96.05	29 s
Momentum			
No	Momentum	Test accuracy (%)	Average time per step (second)
1	0.5	95.39	29 s
2	0.7	98.03	28 s
3	0.9	98.68	30 s
Number of heads			
No	No. of heads	Test accuracy (%)	Average time per step (second)
1	4	95.39	27 s
2	8	99.34	45 s

In the first experiment, three different activation functions, exponential linear unit (elu), rectified linear unit (relu), and hyperbolic tangent function (tanh), are used and the highest accuracy of 96.71% is attained with the relu activation function. Next, two types of hidden units [32] and [64] are experimented with and a test accuracy of 97.37% is achieved with the [32] hidden unit. In the third experiment, the model is tested with 2, 3, and 4 attention layers and the best accuracy, of 98.03%, is obtained with four layers. Three learning rates are experimented with resulting in a test accuracy of 98.68% with a learning rate of 0.000001. Different combinations of the batch size, momentum, and number of heads are experimented with. The highest accuracy of 99.34% is obtained with a batch size of 64, a momentum of 0.9, and 8 heads in 45 s. The results of the ablation study indicate that the model achieves the highest accuracy with activation function: relu, a hidden unit of [32], 4 attention layers, a learning rate of 0.000001, a batch size of 64, a momentum of 0.9, and 8 heads.

### GAT Model’s Performance at Different Correlation Threshold

The initial graph for the feature Table (445 × 10), excluding unique ID and target column, contains 98,790 node connections. Such complex and large dataset has a potential to limit the model’s performance as it can consist of noisy and redundant information [61]. To identify the optimal correlation threshold, an experiment is carried out, as shown in Table 6, to obtain a graph that captures a significant number of node connections.

**Table 6:** Proposed model’s performance at different thresholds

Correlation threshold	Number of edges	Performance of GAT (%)
No threshold	98,790	96.71
$\ge$ 0.5	90,286	96.71
$\ge$ 0.6	75,696	97.37
$\ge$ 0.7	56,054	99.34
$\ge$ 0.8	48,262	98.68
$\ge$ 0.9	33,526	98.68

The Spearman correlation value determines how strongly two nodes are connected. The GAT model gives an accuracy of 96.71% for both cases of considering all edges and edges with a correlation value of 0.5 or higher, highlighting the impact of the presence of irrelevant edges on the model’s performance. The model has an increasing accuracy of 97.37% for threshold values ≥ 0.6. For the thresholds ≥ 0.8 and ≥ 0.9, the model obtains 98.68% accuracy, as there may present lesser number of redundant edges. This study thus chooses a threshold value of ≥ 0.7 which proves itself an optimal threshold, giving the highest accuracy of 99.34%.

### Performance Analysis of Proposed GAT Model

**Fig. 9:**
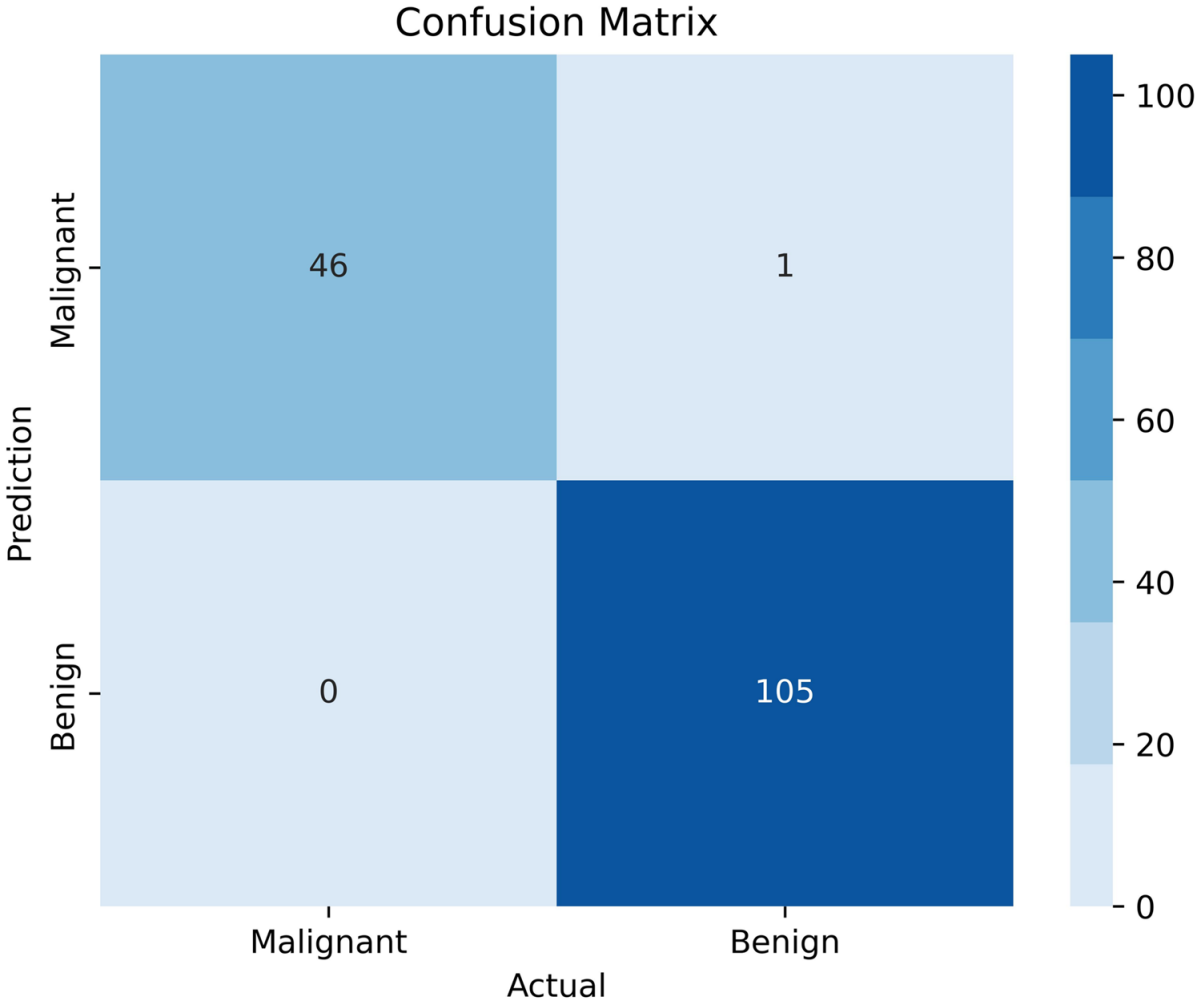
Confusion matrix of proposed model

Several performance metrics, including precision, specificity, sensitivity, NPV, FPR, FDR, FNR, accuracy, F1 score, and MCC, have been calculated to assess the performance of the proposed model. Figure 9 shows the confusion matrix of the model.

The performance metrics are computed using the confusion matrix’s values for TP, TN, FP, and FN. The results are shown in Table 7.

**Table 7:** Evaluation matrix of the GAT model

Performance metric	Results (%)	Performance metric	Results (%)
Training accuracy	99.55	NPV	100
Test accuracy	98.69	FPR	0.94
Validation accuracy	99.34	FDR	2.13
Sensitivity	100	FNR	0
Precision	97.87	F1 score	98.92
Specificity	99.06	MCC	98.46

**Fig. 10:**
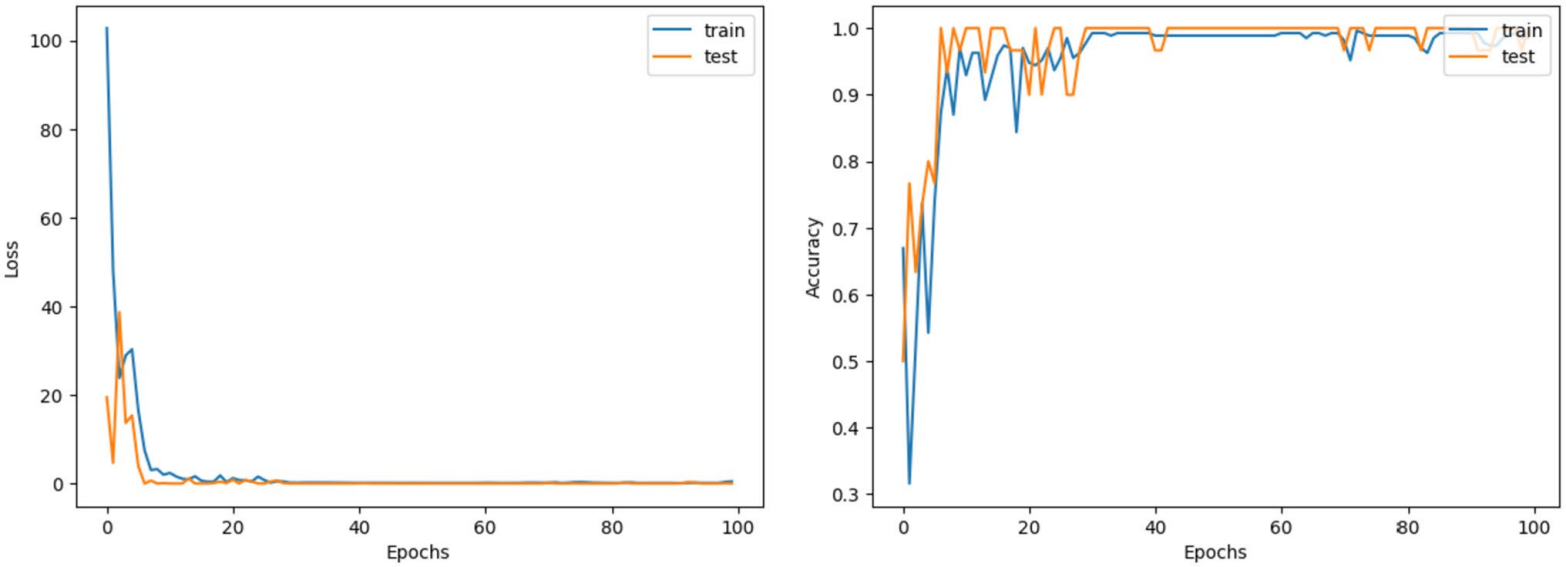
Loss and accuracy curve of the proposed GNN model

The model’s performance metrics are shown in Table 7, where the highest test accuracy of 99.34% is emphasized. Apart from its high accuracy, the model also performs well in terms of other measures: sensitivity, precision, specificity, F1 score, and MCC are all above 97%. The loss and accuracy curves of the model are shown in Fig. 10.

Although there is some variability in the validation accuracy and loss curves, overfitting is not significantly present. This indicates that the proposed model is robust and stable.

### k-Fold Cross Validation

**Fig. 11:**
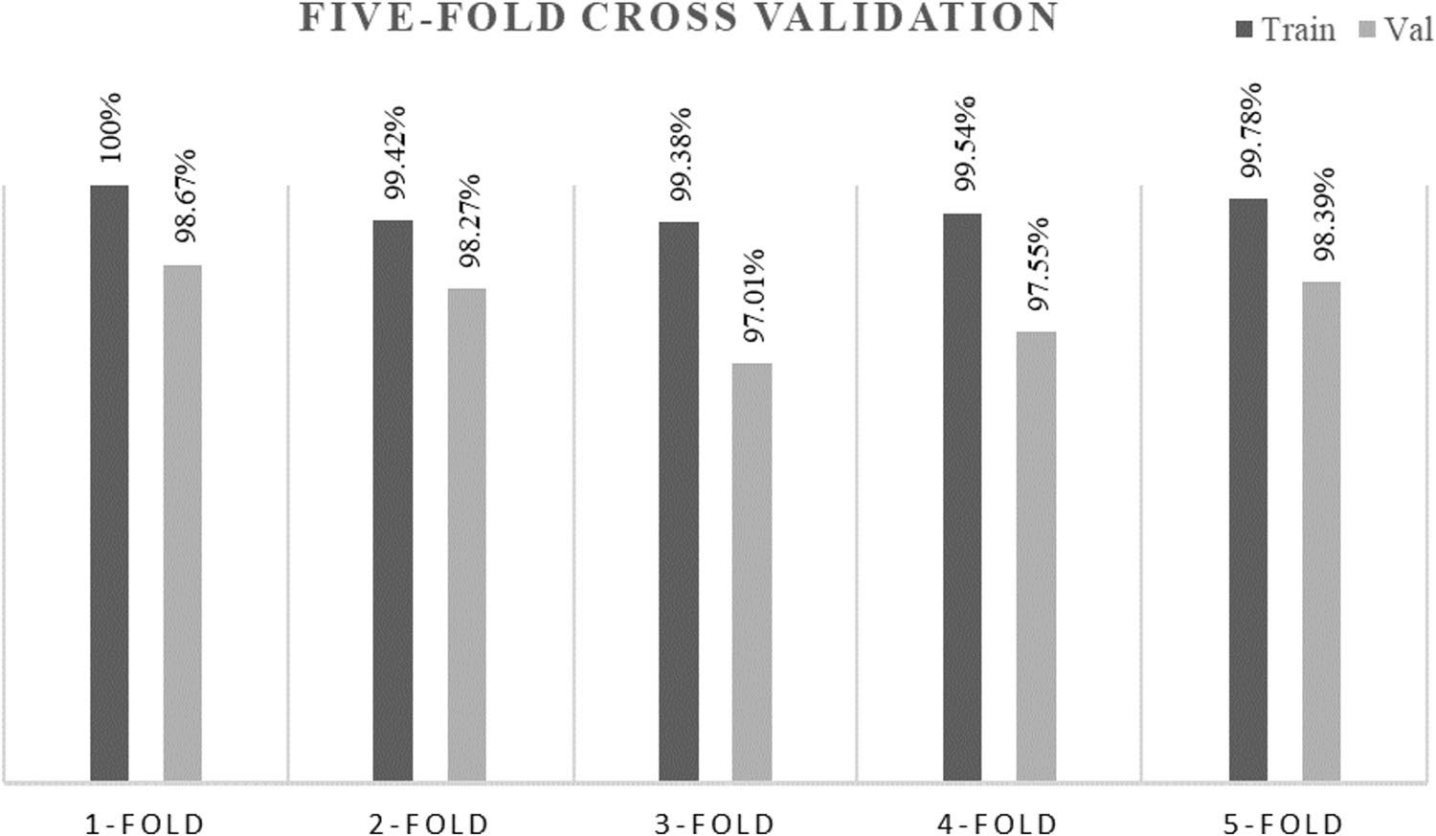
Five-fold cross validation for performance analysis

In k-fold cross validation, the data is shuffled randomly and split into k-groups. In the five-fold cross validation, the dataset is split into five subsets, where four subsets are used for model training and validation is done based on the fifth subset. Using k value 5, a five-fold cross validation is shown in Fig. 11.

It can be observed that, across all folds and iterations in Fig. 11, the GAT model obtains a validation accuracy above 97%, demonstrating its reliable performance in the classification task. This outcome implies that the model is robust enough to operate without overfitting issues.

### Performance Comparison with ML and CNN Models

We have conducted a comparison between our GAT model and ten machine learning models: random forest classifier (RFC), gradient boosting classifier (GBC), extra trees classifier (ETC), bagging classifier (BC), support vector machine (SVM), support vector classifier (SVC), decision tree classifier (DTC), Gaussian naive bias (GNB), k-nearest neighbors (KNN) classifier, logistic regression (LR), and an 1D CNN model. All of these models utilize the same hand-crafted feature set (445 × 11), used for the proposed GAT model’s training and testing purpose. In this case, the “unique ID” column of the feature set (445 × 12) is excluded as it does not contain any mesh-related feature values. Therefore, the models are evaluated on the same subset of data, ensuring consistency in the input of the models throughout the comparison. The results of this comparison are presented in Table 8. This comparison enables us to evaluate the effectiveness and potential superiority of our GAT model compared to the other ML models.

**Table 8:** Comparison with proposed GAT model

Model	Test accuracy
Proposed GAT	99.34%
1D CNN	87.64%
RFC	91.17%
ETC	91.10%
GBC	88.05%
BC	88.05%
SVM	81.77%
SVC	83.58%
KNN	83.58%
DTC	80.59%
LR	79.85%
GNB	73.13%

The results presented in Table 8 indicate that, in comparison to our proposed model, the performance of ML models and 1D CNN model is significantly lower. The 1D CNN model achieves an accuracy of 87.64%, while the accuracy of ML models ranges from 73 to 91%. The substantial performance difference between the proposed model and traditional ML models can be attributed to several factors. ML models often lack the capacity to comprehend the intricate data relationships in small datasets with limited details [62]. Due to the confinement, it can be challenging to obtain consistent data patterns during the training process, which may result in issues such as overfitting, underfitting, and ultimately lower performance [63, 64].

On the other hand, with the graph data representation, the proposed GAT model can significantly understand the intricate data patterns. As described in the “Graph Dataset Generation” section, graph data structures are effective for demonstrating the complex relationships that exist between various data points [65], which is particularly beneficial when working with small datasets. The proposed GAT model utilizes the complex graph data structure and avoids being influenced by noise or irrelevant features by discovering the most important neighborhood connection and focusing on relevant information to gain the remarkable performance.

The results demonstrate that the proposed model outperforms all ML models and the 1D CNN model, indicating its superior ability to learn and make accurate predictions by comprehending data relationships within a graph format as capturing significant patterns is crucial for achieving high performance.

### Comparison with Previous Studies

In this section, a comparison table is presented which includes the key information of each relevant research study compared to our methodology. Authors, year of publication, methodology, and results are shown in Table 9.

**Table 9:** Comparison with previous literature

Authors	Year	Methodology/model	Result (test accuracy)
Qawqzeh et al. [66]	2023	RandomForest, DecisionTree, AdaBoost, and GradientBoosting and modified pcaAdaBoost classifier	97.48%
Luo et al. [67]	2023	Cross-semantic human–machine knowledge fusion and spatial attention	91.07%
He et al. [68]	2023	HCTNet, a hybrid CNN-transformer network	96.94%
Chen et al. [69]	2023	Multi-channel fusion for color conversion, ResNet50-based feature extraction with adaptive spatial feature fusion (ASFF), shallow local binary pattern (LBP) texture features for fusion	96.91%
Ragab et al. [70]	2022	Ensemble Deep-Learning-Enabled Clinical Decision Support System (EDLCDS-BCDC)	96.75%
Sun et al. [71]	2020	CNN and DenseNet	96.20%
Zeimarani et al. [72]	2020	CNN classification with and without augmentation	96%
Zhou et al. [73]	2018	CNN for accurate classification and automatic feature extraction	95.7%

From Table 9, it can be observed that the accuracies of prior studies are between 91 and 97%. The studies are performed with 2D images, where important information such as volume and curvature of breast tumors cannot be assessed. Most authors used CNN and deep learning models, which means that the time complexity of their classification task is much higher. Consequently, our proposed approach demonstrates efficiency and reliability with higher test accuracy and lower computational complexity.

## Discussion

A novel method for classifying diseases is to create a point cloud from a 2D image and then generate a 3D mesh. This may provide more valuable information that can help significantly with precise diagnosis. This study involves a novel approach in the classification of breast cancer using ultrasound images. In this regard, a 2D image is converted into a 3D representation, enabling an in-depth analysis of the underlying structures and features of breast tumor. 3D mesh features are extracted to describe the tumor pattern more precisely. The features are clinically relevant representing tumor characteristic in a more informative way and are used for graph-based classification with a node-to-node relationship. The classification using the GAT model allows to ingrate the graph representation of the features which eventually contributes on increasing the classification performance. In future study, we aim to utilize a larger breast cancer dataset. The proposed approach can be evaluated using a real-world dataset. In this way, an automated method can be proposed which can assist the radiologist in the real-world diagnosis system. In addition, some other classes of breast cancer can be explored to discover more in-depth knowledge regarding tumor pattern. In this way, an automated analysis of breast cancer progression can be conducted.

## Conclusion

In this paper, we have presented a classification of breast tumors from ultrasound images by applying 3D mesh reconstruction from breast ROI 2D image. The mesh is generated employing point-e network where a point cloud is generated from the 2D image. Utilizing the dense point cloud, a 3D triangular mesh is generated. In order to obtain useful information for the classification task, ten mesh features are extracted. In addition, two feature selection techniques, ANOVA and MRMR, are done to determine the features that strongly influence the prediction of the target class. It is found that all ten features are robust. Furthermore, with the ten features, a graph dataset is generated. Then, the graph dataset is fed into the GAT model, resulting in an accuracy of 97.1% was achieved. To enhance the GAT model’s performance, an ablation study is conducted experimenting with seven parameters. The modified GAT model records an accuracy of 99.34%, which validates that the model is robust enough to classify meshes of breast ROI. To conclude, this study demonstrates that the impact of 3D mesh, appropriate feature extraction, and graph-based model can greatly revolutionize the breast tumor classification task.

## Data Availability

The dataset is publicly available and free. The link is attached. https://scholar.cu.edu.eg/?q=afahmy/pages/dataset.

## References

[1] Wang Z, Li M, Wang H, Jiang H, Yao Y, Zhang H, et al. Breast Cancer Detection Using Extreme Learning Machine Based on Feature Fusion with CNN Deep Features. IEEE Access 2019;7:105146–58. 10.1109/ACCESS.2019.2892795.

[2] Lu SY, Wang SH, Zhang YD. BCDNet: An Optimized Deep Network for Ultrasound Breast Cancer Detection. IRBM 2023;44. 10.1016/j.irbm.2023.100774.

[3] Huang Q, Wang D, Lu Z, Zhou S, Li J, Liu L, et al. A novel image-to-knowledge inference approach for automatically diagnosing tumors. Expert Syst Appl 2023;229. 10.1016/j.eswa.2023.120450.

[4] Boumaraf S, Liu X, Ferkous C, Ma X. A New Computer-Aided Diagnosis System with Modified Genetic Feature Selection for BI-RADS Classification of Breast Masses in Mammograms. Biomed Res Int 2020;2020. 10.1155/2020/7695207. 10.1155/2020/7695207 PMC7238352 32462017

[5] Karthik R, Menaka R, Siddharth MV. Classification of breast cancer from histopathology images using an ensemble of deep multiscale networks. Biocybern Biomed Eng 2022;42:963–76. 10.1016/j.bbe.2022.07.006.

[6] Atrey K, Singh BK, Bodhey NK, Bilas Pachori R. Mammography and ultrasound based dual modality classification of breast cancer using a hybrid deep learning approach. Biomed Signal Process Control 2023;86. 10.1016/j.bspc.2023.104919.

[7] Chen G, Dai Y, Zhang J. RRCNet: Refinement residual convolutional network for breast ultrasound images segmentation. Eng Appl Artif Intell 2023;117. 10.1016/j.engappai.2022.105601.

[8] Szymoniak-Lipska M, Polańska A, Jenerowicz D, Lipski A, Żaba R, Adamski Z, et al. High-Frequency Ultrasonography and Evaporimetry in Non-invasive Evaluation of the Nail Unit. Front Med (Lausanne) 2021;8. 10.3389/fmed.2021.686470. 10.3389/fmed.2021.686470 PMC8236586 34195212

[9] Zhang H, Han L, Chen K, Peng Y, Lin J. Diagnostic Efficiency of the Breast Ultrasound Computer-Aided Prediction Model Based on Convolutional Neural Network in Breast Cancer. J Digit Imaging 2020;33:1218–23. 10.1007/s10278-020-00357-7. 32519253 10.1007/s10278-020-00357-7 PMC7572988

[10] Zhang X, Cui H, Hu N, Han P, Fan W, Wang P, et al. Correlation of androgen receptor with ultrasound, clinicopathological features and clinical outcomes in breast cancer. Insights Imaging 2023;14:46. 10.1186/s13244-023-01387-9. 36929229 10.1186/s13244-023-01387-9 PMC10020396

[11] Meng H, Liu X, Niu J, Wang Y, Liao J, Li Q, et al. DGANet: A Dual Global Attention Neural Network for Breast Lesion Detection in Ultrasound Images. Ultrasound Med Biol 2023;49:31–44. 10.1016/j.ultrasmedbio.2022.07.006. 36202677 10.1016/j.ultrasmedbio.2022.07.006

[12] Mendelson EB, Böhm-Vélez M, Berg WA, Whitman GJ, Madjar H, Rizzatto G, et al. ACR BI-RADS® Ultrasound 2013 As of 12/05/2013. n.d.

[13] Guo R, Lu G, Qin B, Fei B. Ultrasound Imaging Technologies for Breast Cancer Detection and Management: A Review. Ultrasound Med Biol 2018;44:37–70. 10.1016/j.ultrasmedbio.2017.09.012. 29107353 10.1016/j.ultrasmedbio.2017.09.012 PMC6169997

[14] Maken P, Gupta A. 2D-to-3D: A Review for Computational 3D Image Reconstruction from X-ray Images. Archives of Computational Methods in Engineering 2023;30:85–114. 10.1007/s11831-022-09790-z.

[15] Ahmedt-Aristizabal D, Armin MA, Denman S, Fookes C, Petersson L. Graph-Based Deep Learning for Medical Diagnosis and Analysis: Past, Present and Future 2021. 10.3390/s21144758. 10.3390/s21144758 PMC8309939 34300498

[16] Nichol A, Jun H, Dhariwal P, Mishkin P, Chen M. Point-E: A System for Generating 3D Point Clouds from Complex Prompts 2022.

[17] Aljuaid H, Alturki N, Alsubaie N, Cavallaro L, Liotta A. Computer-aided diagnosis for breast cancer classification using deep neural networks and transfer learning. Comput Methods Programs Biomed 2022;223. 10.1016/j.cmpb.2022.106951. 10.1016/j.cmpb.2022.106951 35767911

[18] Chattopadhyay S, Dey A, Singh PK, Sarkar R. DRDA-Net: Dense residual dual-shuffle attention network for breast cancer classification using histopathological images. Comput Biol Med 2022;145. 10.1016/j.compbiomed.2022.105437. 10.1016/j.compbiomed.2022.105437 35339096

[19] Abunasser BS, AL-Hiealy MRJ, Zaqout IS, Abu-Naser SS. Convolution Neural Network for Breast Cancer Detection and Classification Using Deep Learning. Asian Pacific Journal of Cancer Prevention 2023;24:531–44. 10.31557/APJCP.2023.24.2.531. 10.31557/APJCP.2023.24.2.531 PMC10162639 36853302

[20] Jabeen K, Khan MA, Balili J, Alhaisoni M, Almujally NA, Alrashidi H, et al. BC2NetRF: Breast Cancer Classification from Mammogram Images Using Enhanced Deep Learning Features and Equilibrium-Jaya Controlled Regula Falsi-Based Features Selection. Diagnostics 2023;13. 10.3390/diagnostics13071238. 10.3390/diagnostics13071238 PMC10093018 37046456

[21] Obayya M, Maashi MS, Nemri N, Mohsen H, Motwakel A, Osman AE, et al. Hyperparameter Optimizer with Deep Learning-Based Decision-Support Systems for Histopathological Breast Cancer Diagnosis. Cancers (Basel) 2023;15. 10.3390/cancers15030885. 10.3390/cancers15030885 PMC9913140 36765839

[22] Yao MMS, Du H, Hartman M, Chan WP, Feng M. End-to-End Calcification Distribution Pattern Recognition for Mammograms: An Interpretable Approach with GNN. Diagnostics 2022;12. 10.3390/diagnostics12061376. 10.3390/diagnostics12061376 PMC9222096 35741186

[23] Sun W, Zhao Y, Liu J, Zheng G. LatentPCN: latent space-constrained point cloud network for reconstruction of 3D patient-specific bone surface models from calibrated biplanar X-ray images. Int J Comput Assist Radiol Surg 2023;18:989–99. 10.1007/s11548-023-02877-3. 37027083 10.1007/s11548-023-02877-3

[24] Jia H, Tang H, Ma G, Cai W, Huang H, Zhan L, et al. A convolutional neural network with pixel-wise sparse graph reasoning for COVID-19 lesion segmentation in CT images. Comput Biol Med 2023;155. 10.1016/j.compbiomed.2023.106698. 10.1016/j.compbiomed.2023.106698 PMC9942482 36842219

[25] Liu Z, Sun Y, Ma A, Wang X, Xu D, Spakowics D, et al. An explainable graph neural framework to identify cancer-associated intratumoral microbial communities n.d. 10.1101/2023.04.16.537088. 10.1002/advs.202403393 PMC11538693 39225619

[26] Laumer F, Amrani M, Manduchi L, Beuret A, Rubi L, Dubatovka A, et al. Weakly supervised inference of personalized heart meshes based on echocardiography videos. Med Image Anal 2023;83. 10.1016/j.media.2022.102653. 10.1016/j.media.2022.102653 36327655

[27] Hu M, Wang J, Chang C-W, Liu T, Yang X. Ultrasound breast tumor detection based on vision graph neural network, SPIE-Intl Soc Optical Eng; 2023, p. 34. 10.1117/12.2654077.

[28] Ma L, Lian C, Kim D, Xiao D, Wei D, Liu Q, et al. Bidirectional prediction of facial and bony shapes for orthognathic surgical planning. Med Image Anal 2023;83. 10.1016/j.media.2022.102644. 10.1016/j.media.2022.102644 PMC10445637 36272236

[29] Al-Dhabyani W, Gomaa M, Khaled H, Fahmy A. Dataset of breast ultrasound images. Data Brief 2020;28. 10.1016/j.dib.2019.104863. 10.1016/j.dib.2019.104863 PMC6906728 31867417

[30] Wei Y, Vosselman G, Yang MY. BuilDiff: 3D Building Shape Generation using Single-Image Conditional Point Cloud Diffusion Models. n.d.

[31] Ubor Ladický L’, Zürich E, Saurer O, Jeong S, Maninchedda F, Pollefeys M. From Point Clouds to Mesh using Regression. n.d.

[32] Zhang J, Zhong D, Wang L. A Two‐Step Surface Reconstruction Method Using Signed Marching Cubes. Applied Sciences (Switzerland) 2022;12. 10.3390/app12041792.

[33] Ayana G, Dese K, Raj H, Krishnamoorthy J, Kwa T. De-Speckling Breast Cancer Ultrasound Images Using a Rotationally Invariant Block Matching Based Non-Local Means (RIBM-NLM) Method. Diagnostics 2022;12. 10.3390/diagnostics12040862. 10.3390/diagnostics12040862 PMC9030862 35453909

[34] Chen H, Zuo Y, Tong Y, Zhu L. 3D point cloud generation reconstruction from single image based on image retrieval. Results in Optics 2021;5. 10.1016/j.rio.2021.100124.

[35] Rock J, Gupta T, Thorsen J, Gwak J, Shin D, Hoiem D. Completing 3D Object Shape from One Depth Image. n.d.

[36] Fu K, Peng J, He Q, Zhang H. Single image 3D object reconstruction based on deep learning: A review. Multimed Tools Appl 2021;80:463–98. 10.1007/s11042-020-09722-8.

[37] Badki A, Gallo O, Kautz J, Sen P. Meshlet Priors for 3D Mesh Reconstruction. n.d.

[38] Lv C, Lin W, Zhao B. Voxel Structure-Based Mesh Reconstruction from a 3D Point Cloud. IEEE Trans Multimedia 2022;24:1815–29. 10.1109/TMM.2021.3073265.

[39] Maruyama T, Hayashi N, Sato Y, Hyuga S, Wakayama Y, Watanabe H, et al. Comparison of medical image classification accuracy among three machine learning methods. J Xray Sci Technol 2018;26:885–93. 10.3233/XST-18386. 30223423 10.3233/XST-18386

[40] Institute of Electrical and Electronics Engineers, IEEE Engineering in Medicine and Biology Society, IEEE Signal Processing Society. IEEE ISBI 2020 International Symposium on Biomedical Imaging: 2020 symposium proceedings : April 3–7, 2020, Iowa City, Iowa. n.d.

[41] Ternifi R, Wang Y, Gu J, Polley EC, Carter JM, Pruthi S, et al. Ultrasound high-definition microvasculature imaging with novel quantitative biomarkers improves breast cancer detection accuracy. Eur Radiol 2022;32:7448–62. 10.1007/s00330-022-08815-2. 35486168 10.1007/s00330-022-08815-2 PMC9616967

[42] Weinmann M, Jutzi B, Hinz S, Mallet C. Semantic point cloud interpretation based on optimal neighborhoods, relevant features and efficient classifiers. ISPRS Journal of Photogrammetry and Remote Sensing 2015;105:286–304. 10.1016/j.isprsjprs.2015.01.016.

[43] Hansen SS, Ernstsen VB, Andersen MS, Al-Hamdani Z, Baran R, Niederwieser M, et al. Classification of boulders in coastal environments using random forest machine learning on topo-bathymetric lidar data. Remote Sens (Basel) 2021;13. 10.3390/rs13204101.

[44] Hu Z, Tang J, Zhang P, Jiang J. Deep learning for the identification of bruised apples by fusing 3D deep features for apple grading systems. Mech Syst Signal Process 2020;145. 10.1016/j.ymssp.2020.106922.

[45] Huo Y, Wang T, Li H, Zhang Y, Li X, Liu B, et al. Delaunay Mesh Construction and Simplification with Feature Preserving Based on Minimal Volume Destruction. Applied Sciences (Switzerland) 2022;12. 10.3390/app12041831.

[46] Limkin EJ, Reuzé S, Carré A, Sun R, Schernberg A, Alexis A, et al. The complexity of tumor shape, spiculatedness, correlates with tumor radiomic shape features. Sci Rep 2019;9. 10.1038/s41598-019-40437-5. 10.1038/s41598-019-40437-5 PMC6416263 30867443

[47] Wei M, Du Y, Wu X, Su Q, Zhu J, Zheng L, et al. A Benign and Malignant Breast Tumor Classification Method via Efficiently Combining Texture and Morphological Features on Ultrasound Images. Comput Math Methods Med 2020;2020. 10.1155/2020/5894010. 10.1155/2020/5894010 PMC7547332 33062038

[48] Mendes J, Domingues J, Aidos H, Garcia N, Matela N. AI in Breast Cancer Imaging: A Survey of Different Applications. J Imaging 2022;8. 10.3390/jimaging8090228. 10.3390/jimaging8090228 PMC9502309 36135394

[49] Hassanien MA, Singh VK, Puig D, Abdel-Nasser M. Predicting Breast Tumor Malignancy Using Deep ConvNeXt Radiomics and Quality-Based Score Pooling in Ultrasound Sequences. Diagnostics 2022;12. 10.3390/diagnostics12051053. 10.3390/diagnostics12051053 PMC9139635 35626208

[50] Tang Y, Liang M, Tao L, Deng M, Li T. Machine learning–based diagnostic evaluation of shear-wave elastography in BI-RADS category 4 breast cancer screening: a multicenter, retrospective study. Quant Imaging Med Surg 2022;12:1223–34. 10.21037/qims-21-341. 10.21037/qims-21-341 PMC8739129 35111618

[51] Suresh S, Newton DT, Everett TH, Lin G, Duerstock BS. Feature Selection Techniques for a Machine Learning Model to Detect Autonomic Dysreflexia. Front Neuroinform 2022;16. 10.3389/fninf.2022.901428. 10.3389/fninf.2022.901428 PMC9416695 36033642

[52] Saeys Y, Inza I, Larrañaga P. A review of feature selection techniques in bioinformatics. Bioinformatics 2007;23:2507–17. 10.1093/bioinformatics/btm344. 17720704 10.1093/bioinformatics/btm344

[53] Aggarwal N, Shukla U, Saxena GJ, Rawat M, Bafila AS, Singh S, et al. Mean based relief: An improved feature selection method based on ReliefF. Applied Intelligence 2023. 10.1007/s10489-023-04662-w.

[54] Ricotti V, Kadirvelu B, Selby V, Festenstein R, Mercuri E, Voit T, et al. Wearable full-body motion tracking of activities of daily living predicts disease trajectory in Duchenne muscular dystrophy. Nat Med 2023;29:95–103. 10.1038/s41591-022-02045-1. 36658421 10.1038/s41591-022-02045-1 PMC9873561

[55] Ali NM, Farouk AIB, Haruna SI, Alanazi H, Adamu M, Ibrahim YE. Feature selection approach for failure mode detection of reinforced concrete bridge columns. Case Studies in Construction Materials 2022;17. 10.1016/j.cscm.2022.e01383.

[56] Jabeen K, Khan MA, Alhaisoni M, Tariq U, Zhang YD, Hamza A, et al. Breast Cancer Classification from Ultrasound Images Using Probability‐Based Optimal Deep Learning Feature Fusion. Sensors 2022;22. 10.3390/s22030807. 10.3390/s22030807 PMC8840464 35161552

[57] Ahmedt-Aristizabal D, Armin MA, Denman S, Fookes C, Petersson L. A survey on graph-based deep learning for computational histopathology. Computerized Medical Imaging and Graphics 2022;95. 10.1016/j.compmedimag.2021.102027. 10.1016/j.compmedimag.2021.102027 34959100

[58] Duvenaud D, Maclaurin D, Aguilera-Iparraguirre J, Gómez-Bombarelli R, Hirzel T, Aspuru-Guzik A, et al. Convolutional Networks on Graphs for Learning Molecular Fingerprints. n.d.

[59] Veličković P, Cucurull G, Casanova A, Romero A, Liò P, Bengio Y. Graph Attention Networks 2017.

[60] Mohammed Alsumaidaee YA, Yaw CT, Koh SP, Tiong SK, Chen CP, Yusaf T, et al. Detection of Corona Faults in Switchgear by Using 1D-CNN, LSTM, and 1D-CNN-LSTM Methods. Sensors 2023;23. 10.3390/s23063108. 10.3390/s23063108 PMC10059847 36991819

[61] Abadal S, Jain A, Guirado R, López-Alonso J, Alarcón E. Computing Graph Neural Networks: A Survey from Algorithms to Accelerators. ACM Comput Surv 2022;54. 10.1145/3477141.

[62] Meng X, Zou T. Clinical applications of graph neural networks in computational histopathology: A review. Comput Biol Med 2023;164. 10.1016/j.compbiomed.2023.107201. 10.1016/j.compbiomed.2023.107201 37517325

[63] Althnian A, AlSaeed D, Al-Baity H, Samha A, Dris A Bin, Alzakari N, et al. Impact of dataset size on classification performance: An empirical evaluation in the medical domain. Applied Sciences (Switzerland) 2021;11:1–18. 10.3390/app11020796.

[64] Xu P, Ji X, Li M, Lu W. Small data machine learning in materials science. NPJ Comput Mater 2023;9. 10.1038/s41524-023-01000-z.

[65] Ma T, Pan Q, Wang H, Shao W, Tian Y, Al-Nabhan N. Graph classification algorithm based on graph structure embedding. Expert Syst Appl 2020;161. 10.1016/j.eswa.2020.113715.

[66] Qawqzeh YK, Alourani A, Ghwanmeh S. An improved breast cancer classification method using an enhanced adaboost classifier. vol. 14. n.d.

[67] Luo Y, Lu Z, Liu L, Huang Q. Deep fusion of human-machine knowledge with attention mechanism for breast cancer diagnosis. Biomed Signal Process Control 2023;84. 10.1016/j.bspc.2023.104784.

[68] He Q, Yang Q, Xie M. HCTNet: A hybrid CNN-transformer network for breast ultrasound image segmentation. Comput Biol Med 2023;155. 10.1016/j.compbiomed.2023.106629. 10.1016/j.compbiomed.2023.106629 36787669

[69] Chen H, Ma M, Liu G, Wang Y, Jin Z, Liu C. Breast Tumor Classification in Ultrasound Images by Fusion of Deep Convolutional Neural Network and Shallow LBP Feature. J Digit Imaging 2023;36:932–46. 10.1007/s10278-022-00711-x. 36720840 10.1007/s10278-022-00711-x PMC10287618

[70] Ragab M, Albukhari A, Alyami J, Mansour RF. Ensemble Deep-Learning-Enabled Clinical Decision Support System for Breast Cancer Diagnosis and Classification on Ultrasound Images. Biology (Basel) 2022;11. 10.3390/biology11030439. 10.3390/biology11030439 PMC8945718 35336813

[71] Sun Q, Lin X, Zhao Y, Li L, Yan K, Liang D, et al. Deep Learning vs. Radiomics for Predicting Axillary Lymph Node Metastasis of Breast Cancer Using Ultrasound Images: Don’t Forget the Peritumoral Region. Front Oncol 2020;10. 10.3389/fonc.2020.00053. 10.3389/fonc.2020.00053 PMC7006026 32083007

[72] Zeimarani B, Costa MGF, Nurani NZ, Bianco SR, De Albuquerque Pereira WC, Filho CFFC. Breast Lesion Classification in Ultrasound Images Using Deep Convolutional Neural Network. IEEE Access 2020;8:133349–59. 10.1109/ACCESS.2020.3010863.

[73] Zhou Y, Xu J, Liu Q, Li C, Liu Z, Wang M, et al. A radiomics approach with CNN for shear-wave elastography breast tumor classification. IEEE Trans Biomed Eng 2018;65:1935–42. 10.1109/TBME.2018.2844188. 29993469 10.1109/TBME.2018.2844188

